# Supplementary material for: Combined Anticancer Effect of Sulfated Laminaran from the Brown Alga Alaria angusta and Polyhydroxysteroid Glycosides from the Starfish Protoreaster lincki on 3D Colorectal Carcinoma HCT 116 Cell Line
Source: Mar Drugs. 2021 Sep 25;19(10):540. doi: 10.3390/md19100540 (PMC8538801; doi:10.3390/md19100540)
Supplement: Supplementary file 1 [file marinedrugs-19-00540-s001.zip › marinedrugs-1371192-supplementary.pdf]

Supporting Information

Combined Anticancer Effect of Sulfated Laminaran from the Brown Alga *Alaria angusta* and Polyhydroxysteroid Glycosides from the Starfish *Protoreaster lincki* on 3D Colorectal Carcinoma HCT 116 Cell Line

Olesya S. Malyarenko *, Timofey V. Malyarenko, Roza V. Usoltseva, Valerii V. Surits, Alla A. Kicha, Natalia V. Ivanchina and Svetlana P. Ermakova

G.B. Elyakov Pacific Institute of Bioorganic Chemistry, Far Eastern Branch of the Russian Academy of Sciences, 159 100-let Vladivostok Ave., Vladivostok 690022, Russia; malyarenko-tv@mail.ru (T.V.M.);
usoltseva-r@yandex.ru (R.V.U.); suritsw@yandex.ru (V.V.S.); kicha@piboc.dvo.ru (A.A.K.); ivanchina@piboc.dvo.ru (N.V.I.); swetlana_e@mail.ru (S.P.E.)
* Correspondence: malyarenko.os@gmail.com; Tel.: +7-8(423)231-07-05; Fax: +8(423)231-40-50

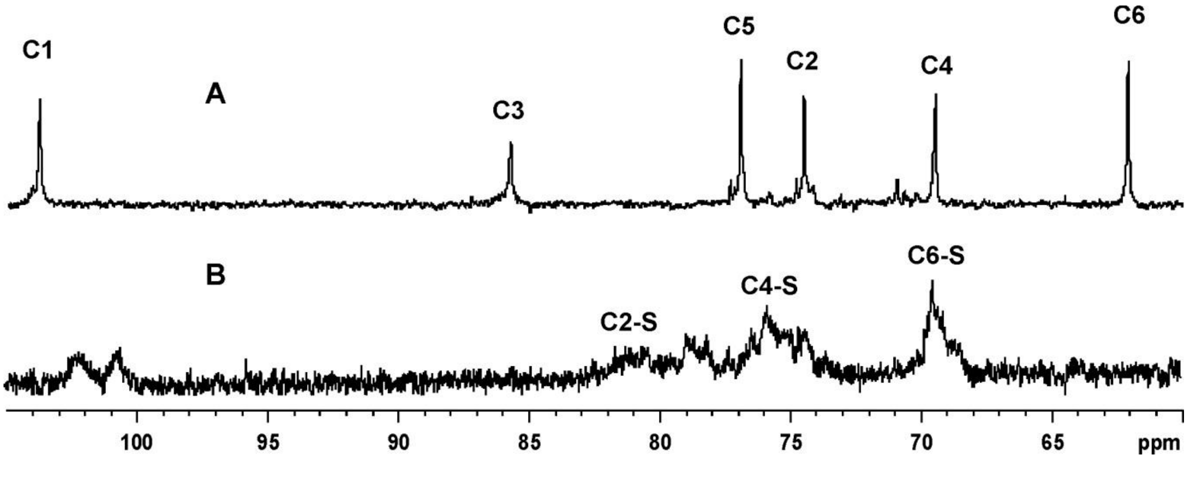

Figure S1. ^{13}C NMR spectra of native **AaL** (A) and sulfated **AaLs** (B) laminarans from *A. angusta*

C1, C2, C3, C4, C5, and C6 – signals of carbons of 1,3-linked β-D-glucose residues in the main chain; S – sulfate groups.

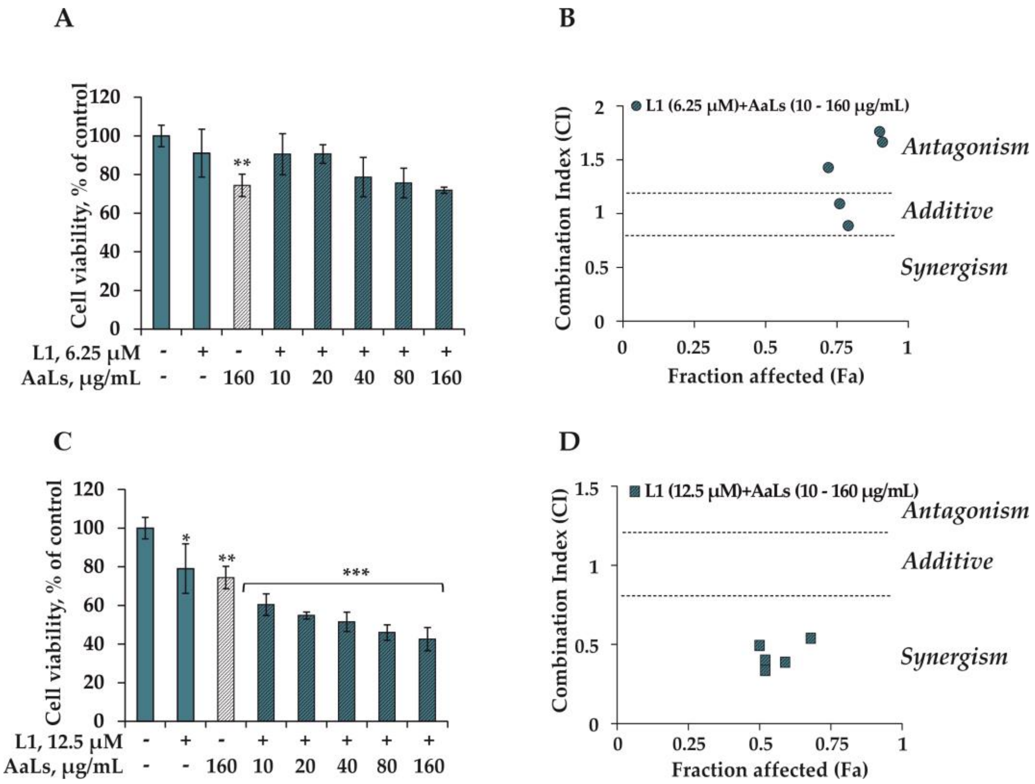

Figure S2. Combined effect of L1 with AaLs on viability of 3D HCT 116 spheroids.

3D HCT 116 spheroids were treated with (A) L1 at 6.25 μM in combination with AaLs (10, 20, 40, 80, 160 μg/mL) or (C) L1 at 12.5 μM in combination with AaLs (10, 20, 40, 80, 160 μg/mL) for 72 h. Cell viability was assessed using the MTS assay. (B, D) Type of combination of L1 with AaLs calculated by by Compusyn software 1.0.1 (ComboSyn, Inc., Paramus, NJ, USA). Combination index (CI) is a quantitative measure of the degree of interaction between different treatments. A CI equal to 0.9–1.1 is considered additive; a CI value of greater than 1.1 represents antagonism; and CI values less than 0.7 denotes synergism. All analyzes were performed in three independent experiments. Results are expressed as mean ± standard deviation (SD). Student's test was used to evaluate data with the following significance level * $p < 0.05$, ** $p < 0.01$, *** $p < 0.001$.
